# Exploratory analyses of cervicovaginal mucus *O-*glycan composition and microbiota profiles in unexplained infertility

**DOI:** 10.1093/glycob/cwag023

**Published:** 2026-03-30

**Authors:** Schahzad Saqib, Dimitrios Latousakis, Seppo Virtanen, Ilkka Kalliala, Tiina Holster, Nathalie Juge, Anne Salonen

**Affiliations:** Human Microbiome Research Program, Faculty of Medicine, University of Helsinki, Haartmaninkatu 8, P.O. Box 63, 00014, Helsinki, Finland; The Food, Microbiome and Health Institute Strategic Programme, Rosalind Franklin Road, Norwich Research Park, Quadram Institute Bioscience, NR4 7UQ, Norwich, United Kingdom; Human Microbiome Research Program, Faculty of Medicine, University of Helsinki, Haartmaninkatu 8, P.O. Box 63, 00014, Helsinki, Finland; Department of Obstetrics and Gynaecology, University of Helsinki and Helsinki University Hospital, Haartmaninkatu 8, P.O. Box 63, 00014, Helsinki, Finland; Human Microbiome Research Program, Faculty of Medicine, University of Helsinki, Haartmaninkatu 8, P.O. Box 63, 00014, Helsinki, Finland; Department of Obstetrics and Gynaecology, University of Helsinki and Helsinki University Hospital, Haartmaninkatu 8, P.O. Box 63, 00014, Helsinki, Finland; Department of Metabolism, Digestion and Reproduction, Faculty of Medicine, Imperial College London, Level 2, Faculty Building, South Kensington Campus, SW7 2AZ, London, United Kingdom; Department of Obstetrics and Gynaecology, University of Helsinki and Helsinki University Hospital, Haartmaninkatu 8, P.O. Box 63, 00014, Helsinki, Finland; The Food, Microbiome and Health Institute Strategic Programme, Rosalind Franklin Road, Norwich Research Park, Quadram Institute Bioscience, NR4 7UQ, Norwich, United Kingdom; Human Microbiome Research Program, Faculty of Medicine, University of Helsinki, Haartmaninkatu 8, P.O. Box 63, 00014, Helsinki, Finland; Department of Bacteriology and Immunology, Faculty of Medicine, University of Helsinki, Haartmaninkatu 8, P.O. Box 63, 00014, Helsinki, Finland

**Keywords:** cervicovaginal mucus, Fucosylation, *O-*glycans/unexplained infertility, vaginal microbiome

## Abstract

In addition to the specific causes of infertility, two components of the vaginal ecosystem, the vaginal microbiota and the cervicovaginal mucus (CVM), may be associated to reduced fecundity and the success of infertility treatments. The aim of this study was to explore the composition of the CVM *O-*glycans and vaginal microbiota in women with unexplained infertility. We collected CVM and vaginal swab samples during medically induced ovulation from 19 women with unexplained infertility. Mucin *O-*glycosylation profiles were generated through Matrix-Assisted Laser Desorption/Ionization Time-Of-Flight (MALDI-ToF) mass spectrometry and taxonomic profiles of the vaginal microbiota through 16S rRNA gene amplicon sequencing. Altogether 57 *O-*glycan structures were identified, dominated by core 1 and 2 structures. A significant proportion, nearly 85%, of the glycans were fucosylated and five structures dominated the profiles, accounting for >50% of the glycans observed in most samples. The vaginal microbiota of the patients was dominated by *Lactobacillus crispatus* (79%), followed by *Lactobacillus jensenii (32%)* and *Lactobacillus iners (*21%) and *Gardnerella vaginalis* (5%, *s*ingle sample). PERMANOVA analysis indicated significant associations between the glycan structures and dominant taxa (q = 0.0011, R2 = 0.37).

This exploratory study provides initial insights into the composition and variation of CVM *O-*glycans in unexplained fertility and in relation to the vaginal microbiota composition, laying a groundwork for future research.

## Introduction

Infertility is the failure to achieve pregnancy after ≥12 months of regular unprotected sexual intercourse and is considered a global health concern affecting up to 17.5% of the adult population (WHO 2023). The main causes of infertility are abnormalities or dysfunctions of the female or male reproductive system, such as ovulatory disorders (i.e. polycystic ovarian syndrome), endometriosis, tubal damage due to sexually transmitted infections (STIs), imbalance of reproductive hormones, sperm disorders (low sperm counts and motility), and the inability to produce sperm, among others (Cui 2010; WHO 2024). However, between 15–30% of couples seeking infertility treatments are diagnosed with “unexplained” infertility, a condition where no obvious abnormalities or dysfunctions within the male or female reproductive systems can be detected (Ray et al. 2012).

The vaginal microbiota is a relative homogenous ecosystem, typically populated with one or few *Lactobacillus* species, specifically *L. crispatus, Lactobacillus iners, Lactobacillus gasseri*, or *Lactobacillus jensenii* (France et al. 2022). *L. crispatus* dominated vaginal microbiota has been repeatedly shown to be associated with vaginal health and positive gynaecological outcomes, including uncomplicated term pregnancies, and higher rate of success in in vitro fertilization (IVF) treatment (Vergaro et al. 2019; Fu et al. 2020; Vainamo et al. 2023). On the other hand, microbial dysbiosis within the vaginal microbiota is characterised as the displacement of *Lactobacillus* species by anaerobic bacteria such as *Gardnerella, Prevotella, Fannyhessia, Sneathia, Veillonella,* and *Streptococcus* spp. This higher diversity and richness in the ecosystem are associated with negative gynaecological outcomes such as bacterial vaginosis (BV) and lower success rate of pregnancies and infertility treatments (Vergaro et al. 2019; Fu et al. 2020; France et al. 2022; Vainamo et al. 2023).

The cervicovaginal mucus (CVM) is a complex, gel-like structure that serves as a protective barrier for the vaginal epithelium and the uterus as well as a selective barrier for sperm (Lacroix et al. 2020). There is also a multifaceted interaction between the CVM and the vaginal microbiota (Vagios and Mitchell 2021). Compositionally, the CVM is >95% water and additionally consists of mucins, nucleic acids, components of the immune system, proteins, fatty acids, and electrolytes (Lacroix et al. 2020). Mucins are glycoproteins decorated with extensive *O-*glycan sugars and are the main structural component of the CVM with three gel forming mucins: MUC2, MUC5AC, and MUC5B and two transmembrane mucins: MUC16 and MUC1 (Andersch-Bjorkman et al. 2007; Lacroix et al. 2020). The volume, viscosity, and texture of the CVM fluctuate with hormonal changes during the menstrual cycle, which is controlled by the proportion of gel-forming and transmembrane mucins and their glycan compositions (Andersch-Bjorkman et al. 2007; Lacroix et al. 2020). This inherently dynamic nature allows the CVM to perform essential biological functions, especially to facilitate the accession of sperm into the uterus during ovulation (Zierden et al. 2023). To date, only few studies have explored the glycosylation patterns of the CVM (Argueso et al. 2002; Andersch-Bjorkman et al. 2007; Wu et al. 2022; Wu et al. 2024). These studies have reported differences in the CVM glycan profiles during ovulation (Argueso et al. 2002; Andersch-Bjorkman et al. 2007). In more recent studies, glycomic analyses of cervicovaginal fluid (CVF) of 10 (Wu et al. 2022) and 40 mainly pregnant women revealed a rich CVF glycome with changes preceding a preterm delivery (Wu et al. 2024).

*O-*glycosylation is the most important structural modification of mammalian mucins. The main building blocks of the *O-*glycans are *N*-acetylgalactosamine (GalNAc), galactose (Gal) and *N*-acetylglucosamine (GlcNAc) chains capped with sialic acid, sulfate and fucose (Fuc). *O-*Glycan structures vary greatly between and within species and anatomical sites, and this variation is known to have significant impact on how commensal and pathogenic bacteria can utilise *O-*glycans as an attachment site and a carbon source in the gut (Bell and Juge 2021; Raba and Luis 2023). In the vaginal ecosystem, BV and other dysbiotic states are characterized by the presence of bacteria with specific glycoside hydrolases, namely fucosidases and sialidases encoded by *Prevotella* and *Gardnerella* species (Segui-Perez et al. 2024) which remove terminal sugar residues, altering mucus structure and integrity of the protective CVM barrier (France et al. 2022; Izadifar et al. 2024). The CVM glycans may serve as a source of nutrition for the vaginal microbiota (Agarwal and Lewis 2021; Vagios and Mitchell 2021; Segui-Perez et al. 2024). However, despite the reciprocal interactions between the CVM and the vaginal microbiota (Vagios and Mitchell 2021), only one prior study profiled both CVM glycans and the vaginal microbiota from the same samples (Wu et al. 2024), in pregnant women at high risk of preterm birth. Here, we study these two components of the cervicovaginal ecosystem during ovulation in women with unexplained infertility.

## Results

### Patient characteristics

A total of 19 women with unexplained infertility were included in the cohort. The mean age of the women were 33 years (range 32 to 35 years). Two of the 19 women (10.53%) become pregnant. The characteristics of the study cohort are summarized in Table 1.

**Table 1:** Summary of background variables collected from patients within this study cohort.

Characteristic	**N = 19** ^a^
**Treatment**	
FSH	3.0 (15.79%)
Letrozole	16.0 (84.21%)
**Age**	33.00 (32.00, 35.00)
**BMI**	25.10 (22.25, 27.10)
**Primary/secondary infertility**	
1	12.0 (63.16%)
2	7.0 (36.84%)
**Prior pregnancies**	
0	12.0 (63.16%)
1	6.0 (31.58%)
2	1.0 (5.26%)
**Prior deliveries**	2.0 (10.53%)
**Prior miscarriages**	4.0 (21.05%)
**Prior abortions**	2.0 (10.53%)
**The thickness of endometrium (mm)**	9.00 (8.20, 10.50)
**Pregnancy**	2.0 (10.53%)

^a^n (%); Median (Q1, Q3)

### The cervicovaginal mucus was predominantly populated by fucosylated core 1 and core 2 glycans

To characterise the mucin glycome in this cohort, mucins were separated from other proteins by gel electrophoresis and western blotting. Each sample was analysed in duplicate. One membrane was probed with an anti-MUC5b primary antibody (Antibodies, Cambridge, UK), followed by an HRP-conjugated anti-rabbit secondary antibody to determine the area where mucins migrated. In each sample, we observed bands corresponding to MUC5b, at MW >260 kDa (Supplementary Fig. 1). The corresponding mucin area was excised from the second gel from each sample, after release by reductive β-elimination, the glycans were desalted and permethylated, prior to MALDI-TOF MS analysis. A total of 57 glycan compositions across all samples was identified (Supplementary Table 1). The mass range of the identified permethylated glycans ranged from 534 Da to 2853 Da. Specifically, we observed sialylated Tn-antigen (Neu5Acα2–6GalNAc, m/z 691) in ~60% of the samples, sialylated or fucosylated core 1 (fucα1-2Galβ1-3GalNAcα-Ser/Thr, m/z 708; Neu5Acα2-3Galβ1-3GalNAcα-Ser/Thr, m/z 895, respectively) in 100% and 67% of the samples, respectively, disialylated core 1 (m/z 1256, Neu5Acα2–3Galβ1–3(Neu5Acα2–6)GalNAc, ID) in 33% of the samples, and core 2 (Galβ1-3(GlcNAcβ1-6)GalNAc) structures in 83% of the samples, as well as further extended core 1 and core 2 structures (fig X), as determined by MS/MS fragmentation experiments (data not shown) of the most abundant glycans, by the presence of characteristic fragments at m/z 520 (reduced GalNAc-Gal fragment). Notably, no core 3 (GlcNAcβ1–3GalNAc) or core 4 (GlcNAcβ1–6(GlcNAcβ1–3)GalNAc) structures could be identified.

**Figure 1:**
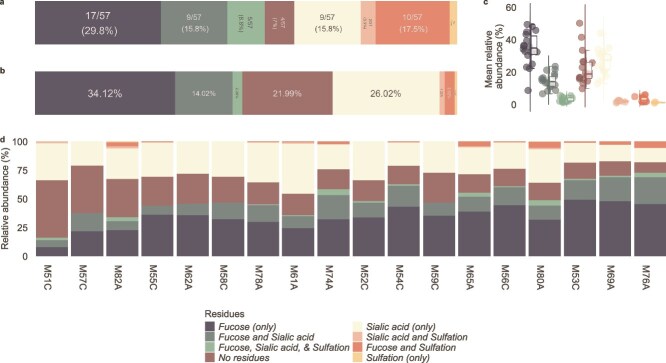
Distribution of terminal residues cervicovaginal mucus glycans structure isolated from the unexplained infertility cohort. a) Tally of glycan structures with each residue type b) mean relative abundance of glycan structures grouped by type of residue c) combined box and violin plots representing the distribution of glycan abundance grouped by type of residue d) bar plot representing the relative abundances of glycan structures grouped by type of residue.

**Figure 2:**
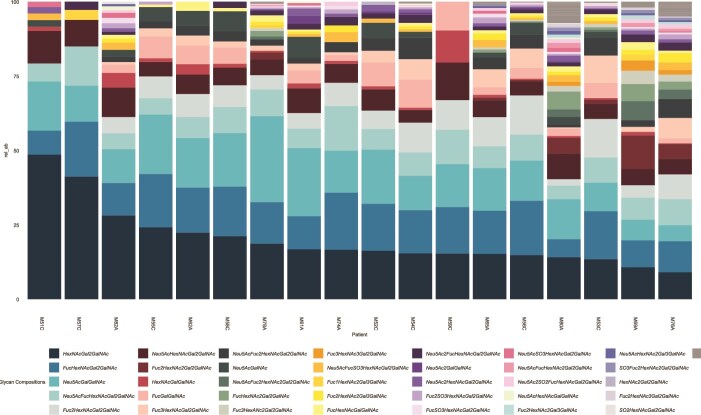
Glycan profiles of the vaginal mucin glycans from the infertility cohort. the first column of the legend shows the most dominant glycan compositions across the samples.

The majority of the identified glycans carried fucose residues, sialic acid residues, sulfate modifications, or a combination of these. Specifically, 71.9% (41/57) of the identified glycans contained fucose residues (29.8% (17/57) had only fucose residues), 43.9% (25/57) contained sialic acid residues (15.8% (9/57) had only sialic acid residues), 15.8% (9/57) identified glycans had both fucose and sialic acid residues, 17.5% (10/57) contained both fucose and sulfate residues, 3.5% (2/57) contained both sialic acid and sulfate residues, and 8.8% (5/57) contained fucose, sialic acid, and sulfate residues. Another 7% (4/57) of the structures contained no capping residues and despite being low in number, made a large proportion of the glycan compositions (21.99%, Figs 1 and 2). Structures containing only fucose or sialic acid residues were the most abundant, with 34.12% and 26.02% mean relative abundance across all samples (Figs 1 and 2). Five glycan compositions dominated the profiles, including a core 2 structure with no capping residues (HexNAcGal2GalNAc), a fucosylated core 2 structure (FucHexNAcGal2GalNAc), a sialylated core 1 structure (Neu5AcGalGalNAc), a core 2 structure with fucose and sialic acid residues (Neu5AcFucHexNAcGal2GalNAc), and a di-fucosylated core 2 structure (Fuc2HexNAcGal2GalNAc). These 5 structures accounted for more than 50% of the total glycans in most samples.

### Vaginal microbiota was characterized by high prevalence and abundance of *lactobacillus* species, primarily *L. crispatus*

**Figure 3:**
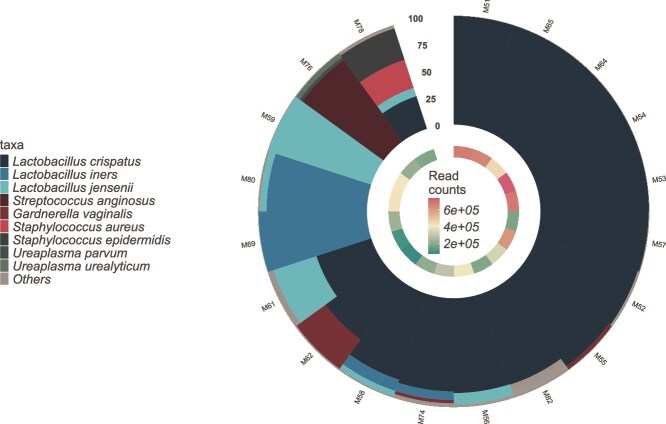
Polar plot representing the sample-wise bacterial profiles observed in the vaginal samples.

*Lactobacillus* species were the most prevalent taxa observed in the study cohort, with *L. crispatus* being the most prevalent (78.9%, 15/19 samples) and abundant (67.8% mean relative abundance) taxon. *L. iners* was observed in 4/19 samples (21%), *L. jensenii* in 31.6% (6/19), and *Gardnerella vaginalis* was observed in a single sample. Other low prevalence pathogens observed included *Staphylococcus aureus, Streptococcus anginosus,* and *Ureaplasma parvum* (Fig. 3)*.*

### Cervicovaginal mucin *O-*glycan profiles in relation to vaginal microbiota composition

**Figure 4:**
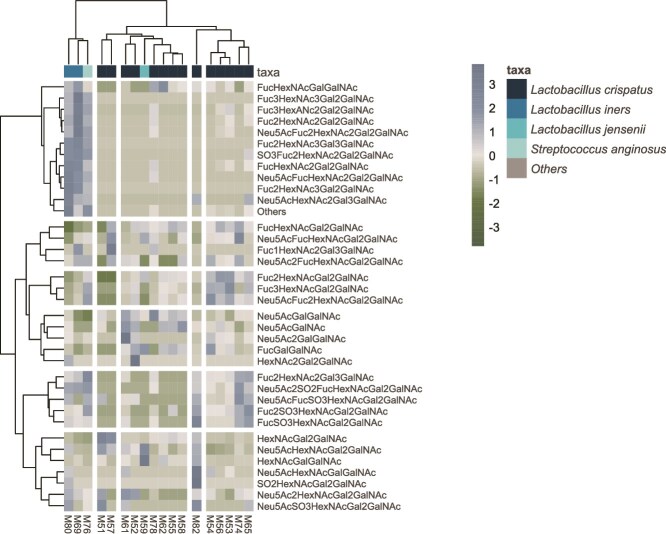
Heatmap illustrating the relative abundances of top 35 mucin O-glycan structures detected in each sample. Column annotations represent the dominant taxon (maximum relative abundance) per sample.

Next, we studied the potential co-variance withing the CVM glycans and with the vaginal microbiota. PERMANOVA analysis revealed significant associations between the glycan structures and dominant taxa (q = 0.0011, R2 = 0.37). Due to the small sample size, we refrained from further statistical analyses but explored the variance through data visualization. In an unsupervised clustering, the glycans formed six clusters based on their abundances across the samples (). The clustering revealed potential patterns in glycan variation: Glycan profiles of the *L. iners* & *S. anginosus*-dominated samples had the most well separated cluster, with enrichment of glycans in the 4^th^ cluster from the top of Fig. 4 driving the separation. Glycan profiles in the *L. crispatus*-dominated samples formed three distinct clusters, the 1^st^ and 6^th^ clusters of glycans being most different.

## Discussion

Here, we present the CVM *O-*glycome of 19 subfertile women using samples collected during ovulation. Overall, the glycome was diverse, with 57 unique glycan compositions identified.

To our knowledge this is the first study profiling the CVM glycome in relation to infertility in humans with a study cohort of subfertile women undergoing fertility treatment. A pioneering study investigating differences between CVM glycans before and during ovulation in 6 healthy women with regular menstrual cycles, identified 50 O-glycan structures (Andersch-Bjorkman et al. 2007). Another recently published study characterises patterns of interactions between vaginal bacteria and CVM glycans and their implications in reproductive outcomes (Tajadura-Ortega et al. 2025). Specifically, it reports that one of the protective effects of *L. crispatus* during pregnancy is through competitive binding to certain glycan structures that are also targeted by pathogenic bacteria such as *Streptococcus agalactiae* (Tajadura-Ortega et al. 2025). Here, we showed that the CVM glycome was dominated by core 1 or core 2 structures, as previously reported for CVM (Argueso et al. 2002; Wu et al. 2024). A majority of core-2 *O-*glycans (45%) followed by core 1 (22%) in sheep CVM samples, studied to understand the biological basis of breed-specific fertility differences (Abril-Parreno et al. 2022). In an earlier human study, ovulatory samples were increased in core 2 glycans compared to samples from the pre- or postovulatory phase (Andersch-Bjorkman et al. 2007). Our samples, all collected during medically induced ovulation, were in line with the sheep study but not with the only prior human study including ovulatory samples.

Interestingly, we identified a similar number of glycan compositions as Andersch-Bjorkman et al (Andersch-Bjorkman et al. 2007). However, we identified a core of up to five dominant glycan compositions accounting for more than 50% of the total glycans, whereas Andersch-Bjorkman et al found a more diverse core glycome. While our study did not include participants as controls, it is tempting to speculate that the differences in glycosylation of mucins may play a role in fertility.

We further observed that the vast majority (84%) of the identified glycans were fucosylated, in line with the earlier study in human samples (Andersch-Bjorkman et al. 2007). Fucosylation of glycans is conferred by fucosyltransferase (FUT) enzymes that are expressed in tissue-specific patterns. *FUT2*, the secretor gene, encodes an α (1,2) fucosyltransferase found particularly in mucosal surfaces secreting mucus, including the genitourinary tract. The abundant fucosylation in our samples primarily indicates activity of *FUT2*. In a mouse model, *fut2* is highly active in the glandular epithelium of the endocervix and strongly regulated by hormonal changes, being most active during the estrous cycle and pregnancy (Domino and Hurd 2004). More specifically, a tissue-specific estrogen-dependency of *fut2* has been documented, showing almost 10-fold fluctuation in the uterine during the estrous cycle, while the Fut2 levels in the colon remained constant (Domino and Hurd 2004). Our findings revealing for the first time the extent of fucosylation of CVM *O-*glycans, warrant further investigations on how CVM fucosylation may differ between infertile and fertile women.

In the gut, bacteria can induce epithelial fucosylation, and fucose is used as an energy source used by many intestinal bacteria (Goto et al. 2014). Lactic acid produced by lactobacilli is known to increase the level of core fucosylation in vaginal epithelial cells (Fan et al. 2021), but there are no prior studies directly assessing the association between fucosylated glycans and the vaginal microbiota. Due to the almost exclusive dominance of lactobacilli in our samples, we could not address the potential impact of microbiota composition on the fucosylation rate.

Sialic acid modifications are relevant for fertility, because they are important modulators of the mucus viscosity and the biochemical interactions with sperm. In sheep, breeds with low fertility had high sialic acid content in cervix coupled with fewer sperm in the cervical crypts following cervical insemination, implying large mucin *O-*glycans may impede sperm migration through the cervix (Richardson et al. 2019). On the other hand, sialic acid of the cervical mucus binds *N*-acetyl glucosamine on the sperm surface with high affinity (Tecle and Gagneux 2015; Ma et al. 2016). A comparative study on *O-*linked glycans in cervical mucus of sheep breeds with high and low fertility showed that, in sheep, higher fucosylation and lower sialylation seemed to be the optimal CVM environment for fertility (Abril-Parreno et al. 2022). The same study reported increased core 4 glycan (GlcNAcβ1–3[GlcNAcβ1–6]GalNAc), core 2 sulfated glycan (Galβ1–3[SO3-GlcNAcβ1–6]GalNAc) and a fucosylated glycan (GlcNAcβ1–3(Fucα1–2Galβ1–3)GalNAc) be related to impaired sperm transport of the CVM, suggesting that increased rather than impaired mucus glycosylation may be related to infertility.

Here, we focused on patients with unexplained infertility where the common causes of infertility had been excluded, with a hypothesis that altered CVM glycan and/or microbiota features more likely play a role compared to women with known etiology of infertility e.g. due to inflammatory, hormonal or anatomical reasons. We found the vast majority, altogether 95% of women had *Lactobacillus* dominant-vaginal microbiota and 79% had *L. crispatus*. Only two (11%) women had *L. iners* in this study population*.* The human vaginal and endometrial microbiota has been studied in relation to fertility by comparing infertile women with mixed etiologies, or in relation to the success of assisted reproductive treatments (Vitale et al. 2021). These studies have mainly reported *Lactobacillus*-dominated microbiota to prevail in fertility and successful pregnancies, and particularly *L. crispatus* playing a central role. The atypically “healthy” microbiota profiles of subfertile women in our cohort are most likely explained by the fact that sampling took place at the peak of ovulation, when oestrogen levels in the circulation are high. Estrogen promotes deposition of glycogen in vaginal epithelium, which in turn promotes *Lactobacillus* growth (Kaur et al. 2020).

To our knowledge, one single study focused on the vaginal microbiota in unexplained infertility. Based on qPCR assessment, infertile women had significantly more often *Lactobacillus*-impaired microbiota (Sezer et al. 2022). This contrasts to studies reporting higher *Lactobacillus*-dominance in infertile women with a history of repeated implantation failure compared to women undergoing their first IVF attempt (Kitaya et al. 2019). Recently, a large study involving almost 1500 infertile women concluded that not only low, but also too high (>90%) abundance of either *L. crispatus* or *L. iners* solely (compared to their co-existence) associated to lower success of becoming pregnant after transfer of fresh or frozen embryos (Wang et al. 2024a). Hence, more research is needed to gain conclusive results. One challenge in interpreting the findings about the role of vaginal microbes in fertility is the substantial heterogeneity of studies, even in the clinical phenotypes, e.g. studies addressing the success of IVF treatment may include patients with both infertility and recurrent miscarriages. Also, the causes of infertility are often multifactorial and diagnostic methods evaluating the underlying etiologies are far from optimal.

Due to the sample size limitation, this exploratory study did not allow statistical inference for the potential associations between the individual CVM glycan structures and the vaginal microbiota, but PERMANOVA and visual inspection of the data clearly indicated that the glycan profiles were not random in relation to the microbiota composition. We also acknowledge that different treatment protocols and length of treatment might potentially cause variations in bacterial and CVM O-glycan profiles, however, due to limited sample size and uneven distribution between sample groups we did not test for potential differences. As the samples were collected during ovulation, the abovementioned homogeneity of the microbiota may overshadow a potentially more pronounced co-variance present outside ovulation. A recent study (Wu et al. 2024) that investigated the correlation between the vaginal microbiota and CFV glycans of pregnant women, also with high estrogen levels, found that paucimannose, high-mannose *N*-glycans and sialylated N-glycans correlated with distinct microbiota profiles, but no correlation between *O-*glycans and vaginal microbiota was reported. In our data, only one sample contained *Gardnerella*, where different species and strains have been shown to possess different virulence factors and varying sialidase activity (Hickey and Forney 2014; Vaneechoutte et al. 2019). A recent study utilising organ-on-a-chip model of human cervix reported *L. crispatus* to significantly increase mucus layer thickness in vitro, but changes in cervical mucus glycans and disruption of the epithelial barrier were only reported following inoculation of the model with *G. vaginalis* (Izadifar et al. 2024)*.* Collectively, these results indicate that larger cohorts with more substantial variation in the vaginal microbiota community types are needed to address the question on the degree of co-variation between the microbiota and *O-*glycan composition of the CVM. Finally, it should be noted that in vivo sampling of the CVM captures not only the intact *O-*glycan structures, but the product of their microbial degradation. Hence, differentiation between intact vs. truncated glycans, if possible, might provide better insights into the utilization patterns of the CVM by the vaginal microbiota.

Only two (10.5%) women in our study cohort became pregnant as the result of the treatment cycle in which the CMV samples were taken. The success rate is in line with the general success rate of IUI combined to OS, which is recommended as a first line treatment for couples with unexplained infertility (Guideline Group on Unexplained et al. 2023). Our study has several limitations. We included a small number of patients and had no control group for subfertile women. A potential control group within an infertility study cohort may include couples with male factor infertility, however, they might also have some underlying causes for infertility. An optimal control group would be fertile women sampled at the same stage of the cycle. The diagnosis of unexplained infertility is generally based on the absence of any abnormalities of the female and male reproductive systems after standard infertility investigations. However, there is no consensual standardization of the diagnostic investigations. Therefore, we cannot exclude that some women in this group may present some mild pathologies in their reproductive systems (i.e. mild endometriosis). Regarding the strengths of our study, all samples were collected at synchronised cycle stage with verified ovulation to exclude individual variation related to sex hormones that are known to affect both the CVM and the vaginal microbiota. Sampling spanning the natural or medically induced hormonal changes during the assisted reproductive treatments (Vainamo et al. 2023) will enable comparative assessment to provide further insights into how hormonal regulation affects the vaginal ecosystem on molecular level.

## Materials and methods

### Study population and sample collection

This study was conducted in the Reproductive Medicine Unit, Helsinki University Hospital (Helsinki, Finland) between December 2020 and August 2021. The study population composed of 19 subfertile women who had unexplained infertility. Inclusion criteria was a failure to achieve a pregnancy after ≥12 months of regular sexual intercourses, age ≤ 40 years, and body mass index (BMI) ≤ 35 kg/m2. Women were treated by intrauterine insemination (IUI) with ovarian stimulation (OS) by letrozole (Letrozol®, *n* = 16) or recombinant follicle stimulating hormone (FSH, Gonal-F®, *n* = 3). This treatment protocol has been recommended as the first line fertility treatment in couples who have unexplained infertility (Guideline Group on Unexplained et al. 2023).

Samples were collected during the treatment cycle at the IUI visit, just before IUI was performed. IUI was scheduled on the same day when the ovulation test detecting a rise in luteinizing hormone in urine was positive in women who ovulated spontaneously (*n* = 16), or the ovulation was triggered with human chorionic gonadotropin (Ovitrelle®, *n* = 3) and IUI was scheduled 36 hours after the trigger. Ovulation was confirmed by intravaginal ultrasound examination. CVM samples were collected during speculum examination by aspirating mucus into 5 mL sterile syringe. Vaginal swab samples were collected with sterile flocked swabs (FLOQSwabs, Copan spa, Italy). Lubricants were not used during sampling. Samples were severed to 1,5 mL Eppendorf tubes which were frozen immediately at −20 °C and further moved to −80 °C within four weeks.

### Cervicovaginal mucus analysis

#### Mucin extraction

A graphical representation of the cervicovaginal mucin analysis workflow is shown in Supplementary Fig. 2. Briefly, to extract the mucins, the mucus samples were resuspended in 1 mL Tris–HCl buffer (200 mM, pH 8), supplemented with 2% SDS, and incubated overnight at 37 °C. Reduction of disulfide bonds was carried out by adding a dithiothreitol solution (final concentration 10 mM) to the samples followed by incubation at 60 °C for 90 min. Iodoacetamide was added to the samples (final concentration 100 mM) to prevent reformation of disulfide bonds and incubated in the dark for an additional 90 min at room temperature. Iodoacetamide was added to the suspension to prevent reformation of disulfide bonds and incubated in the dark for 90 min at room temperature. Next, samples (150 μL) were concentrated using 100 kDa MWCO spin filters (Sigma) as per manufacturer’s instructions. To the retrieved samples, 15 μL of TruPAGE™ LDS sample buffer (Sigma) was added. A 1% agarose solution was prepared in Tris-glycine-SDS (TGS; Biorad) and loaded onto Mini-PROTEAN® Tetra Handcast Systems cassettes (Bio-Rad) as per the manufacturer’s instructions. The samples were heated at 70 °C for 15 min, loaded onto the agarose gels, and electrophoresis was carried out at 100 V for 35 min. The samples were transferred onto an Immobilon Psq PVDF membrane by semi-dry western blot in Tris-glycine buffer (Biorad) at 25 V, 1 A for 60 min using a Trans-Blot® Turbo™ Transfer System at 25 V, 1A, for 60 min. The membranes were retrieved and the region where mucins migrated was sliced individually for each sample and transferred into glass tubes, followed by rinsing with methanol and covered in 250 μl of water.

#### Release of O-glycans and permethylation

To remove the O-glycans from mucin protein backbone, reductive β-elimination reaction was carried out by adding 250 μL of 1 M sodium borohydride solution (NaBH4, dissolved in 0.1 M sodium hydroxide (NaOH)). The samples were incubated at 45 °C overnight in a heating block. The reaction was terminated by addition of 1 mL 5% acetic acid. Desalting columns were assembled by packing a disposable 230 mm glass Pasteur pipette with glass wool to obstruct the flow of the solution and adding Dowex 50 W X8 hydrogen form (H+) beads (mesh 200–400; Sigma). The columns were washed with 3 mL 5% acetic acid, followed by loading of the samples. The fractions were collected by adding another 3 mL acetic acid to the columns and dried overnight in a Genevac vacuum concentrator (Biopharma Group). To remove borate salts, 5% acetic acid in methanol (1 mL) was added to the samples and dried by centrifugal evaporation. The process was repeated 3 times.

To improve efficiency of detection through mass spectrometry, permethylation was carried out on the released *O-*glycans. First, a permethylation solution was prepared combining 200 μL of NaOH and 400 μL of methanol, followed by 4 mL of dry dimethyl sulfoxide (DMSO). The mixture was centrifuged at 4500 rpm for 2 min, and the supernatant (with salts) was discarded, leaving a gel like suspension at the bottom. This step was repeated until the supernatant was clear and free of salts, after which the gel was broken up and suspended in DMSO. To each sample, 100 μL DMSO, 150 μL permethylation solution, and 80 μL iodomethane were added and the samples were agitated on a vortex mixer for 120 min. Next, the reaction was quenched by the addition of 500 μL of H_2_O, yielding a cloudy solution. Excess of iodomethane was removed from the samples with a gentle flow of nitrogen until the samples became clear. The permethylated *O-*glycans were extracted with solid phase extraction. Briefly, the samples were loaded onto hydrophilic–lipophilic balance cartridges attached to a vacuum manifold. The samples were washed three times with 1 mL of water to wash away any salts or contaminants, followed by three washings with 1 mL methanol to elute the permethylated glycans. Samples were dried overnight by centrifugal evaporation.

#### Matrix-assisted laser desorption/ionization time-of-flight (MALDI-ToF) mass spectrometry and annotation of spectra

Once dried, 10 μL of TA50 (0.1% trifluoracetic acid (TFA) and 50% acetonitrile) was added to the samples. A target plate 384 AnchorChip™ (Bruker) was prepared by adding 0.5 μL of super- dihydroxybenzoic acid (Super-DHB) and 0.5 μL of sample. MALDI-ToF mass spectrometry was carried out using an autoflex®Speed MALDI system and the resulting spectra were calibrated and exported through the accompanying software. The spectra were manually inspected, and glycan compositions were identified with glycoworkbench. The final list was manually validated to make sure no peaks went unnoticed. Structures were annotated by applying the “match.closest” function from the MALDIquant R package (Gibb and Strimmer 2012) with a tolerance of 0.3 to identify the closest structure by m/z values. The raw and processed data are available in GlycoPOST (accession number GPST000638).

#### DNA extraction and 16S rRNA gene sequencing

Vaginal swabs were processed for sequencing as described earlier (Virtanen et al. 2019). Briefly, bacterial DNA was extracted through mechanical disruption by a repeated bead beating method, followed by amplification, and index PCR. The targeted V3-V4 16S rRNA gene amplicons were generated with primers 341F 5′-CCTACGGGNGGCWGCAG-3′ and 785Rev 5′-GACTACHVGGGTATCTAATCC-3 (Klindworth et al. 2013) and barcoded using indexing primers with adapters for sequencing on the Element AVITI™ System. The pooled libraries were sequenced with an AVITI™ system using paired end 2 × 300 bp reads at the DNA Sequencing and Genomics Laboratory (BIDGEN), University of Helsinki. Paired end sequencing reads obtained were processed through the dada2 16S rRNA gene amplicon pipeline (v1.16) and the DADA2 R package (v1.32) (Callahan et al. 2016). The amplicon sequencing variants (ASVs) obtained from the dada2 pipeline were assigned species level taxonomic annotations using the taxminer R package (Virtanen et al. 2023).

## Supplementary Material

cwag023_Supplemental_Files

## Data Availability

All R scripts, supplement data, and clinical metadata will be made available on the GitHub repository SchahzadSaqib/O-glycans. Raw sequencing data has been submitted to ENA (PRJEB110423) and will be made public upon acceptance of the manuscript. Glycomics data are accessible on GlycoPOST Watanabe Y, Aoki-Kinoshita KF, Ishihama Y, Okuda S. GlycoPOST realizes FAIR principles for glycomics mass spectrometry data. Nucleic Acids Res. 2021:49(D1):D1523–D1528. https://doi.org/10.1093/nar/gkaa1012 (# GPST000638). Additional information can be made available by the corresponding author upon reasonable request.
