# Supplementary material for: Exploratory analyses of cervicovaginal mucus O-glycan composition and microbiota profiles in unexplained infertility
Source: Glycobiology. 2026 Mar 30;36(5):cwag023. doi: 10.1093/glycob/cwag023 (PMC13082223; doi:10.1093/glycob/cwag023)
Supplement: cwag023_Supplemental_Files [file cwag023_supplemental_files.zip › Supplementary_Material.docx]

**Supplementary Material**

Exploratory analyses of cervicovaginal mucus *O-*glycan composition and microbiota profiles in unexplained infertility

Schahzad Saqib^1^*, Dimitrios Latousakis^2^*, Seppo Virtanen^1,3^, Ilkka Kalliala^1,3,4^, Tiina Holster^3^, Nathalie Juge^2^*^#^, Anne Salonen^1^*^#^

*Equal contribution

^#^Corresponding author

^1^ Human Microbiome Research Program, Faculty of Medicine, University of Helsinki, Helsinki, Finland

^2^ The Food, Microbiome and Health Institute Strategic Programme, Norwich Research Park, Quadram Institute Bioscience, Norwich, UK

^3^ Department of Obstetrics and Gynaecology, University of Helsinki and Helsinki University Hospital, Helsinki, Finland

^4^ Department of Metabolism, Digestion and Reproduction, Faculty of Medicine, Imperial College London, London, United Kingdom

Running title: Mucin *O-*glycan profiles in unexplained infertility

Key words: Cervicovaginal mucus/Fucosylation/*O-*glycans/Unexplained infertility/Vaginal microbiome

Correspondence: Anne Salonen, [anne.salonen@helsinki.fi](mailto:anne.salonen@helsinki.fi), Nathalie Juge,

[nathalie.juge@quadram.ac.uk](mailto:nathalie.juge@quadram.ac.uk)


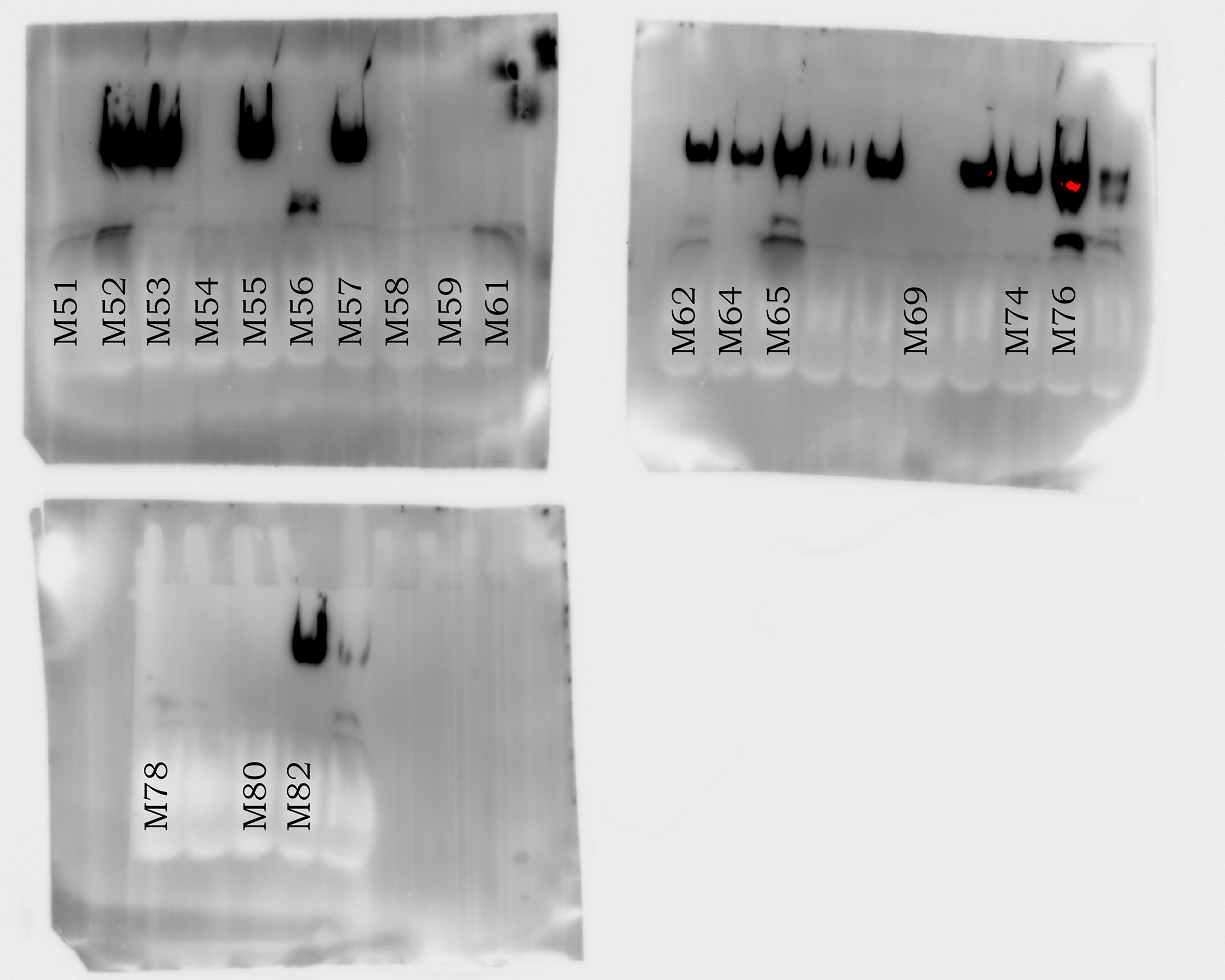


Supplement Figure 1: Gel images for detection of MUC5b


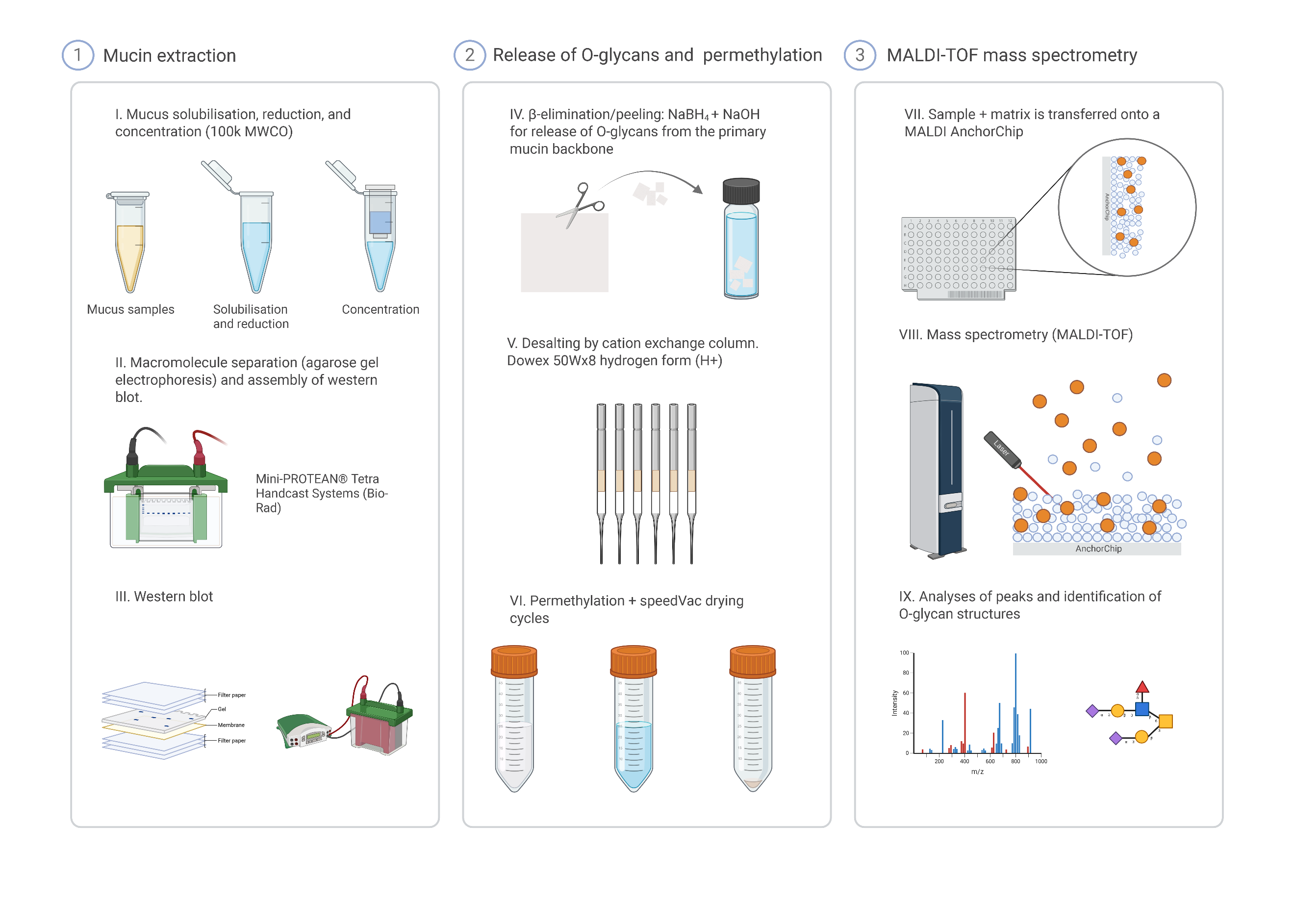


Supplement Figure 2: Cervicovaginal mucus analysis workflow. Created in Biorender.
